# Peripheral vertigo and subsequent risk of depression and anxiety disorders: a prospective cohort study using the UK Biobank

**DOI:** 10.1186/s12916-023-03179-w

**Published:** 2024-02-09

**Authors:** Xiaowan Chen, Dang Wei, Fang Fang, Huan Song, Li Yin, Magnus Kaijser, Tiril Pedersen Gurholt, Ole Andreas Andreassen, Unnur Valdimarsdóttir, Kejia Hu, Maoli Duan

**Affiliations:** 1 Department of Otolaryngology Head and Neck Surgery, the First Hospital of Lanzhou University, Lanzhou, Gansu Province China; 2 Unit of Integrative Epidemiology, Institute of Environmental Medicine, Karolinska Institutet, 171 77 Stockholm, Sweden; 3 Department of Otolaryngology Head and Neck Surgery & Audiology and Neurotology, Karolinska University Hospital, Stockholm, Sweden; 4 Department of Clinical Science, Intervention and Technology, Karolinska Institutet, 171 77 Stockholm, Sweden; 5 West China Biomedical Big Data Center, West China Hospital, Sichuan University, Chengdu, China; 6 Med-X Center for Informatics, Sichuan University, Chengdu, China; 7 Centre of Public Health Sciences, Faculty of Medicine, University of Iceland, Reykjavik, Iceland; 8 Department of Medical Epidemiology and Biostatistics, Karolinska Institutet, Stockholm, Sweden; 9 Department of Neuroradiology, Karolinska University Hospital, Stockholm, Sweden; 10 Norwegian Centre for Mental Disorders Research (NORMENT), Division of Mental Health and Addiction, Institute of Clinical Medicine, Oslo University Hospital &, University of Oslo, Oslo, Norway; 11 Department of Epidemiology, Harvard TH Chan School of Public Health, Harvard University, Boston, MA USA

**Keywords:** Peripheral vertigo, Psychiatric disorders, Fractional anisotropy, Frontal-limbic network

## Abstract

**Background:**

Peripheral vertigo is often comorbid with psychiatric disorders. However, no longitudinal study has quantified the association between peripheral vertigo and risk of psychiatric disorders. Furthermore, it remains unknown how the white matter integrity of frontal-limbic network relates to the putative peripheral vertigo-psychiatric disorder link.

**Methods:**

We conducted a cohort study including 452,053 participants of the UK Biobank with a follow-up from 2006 through 2021. We assessed the risks of depression and anxiety disorders in relation to a hospitalization episode involving peripheral vertigo using Cox proportional hazards models. We also examined the associations of peripheral vertigo, depression, and anxiety with MRI fractional anisotropy (FA) in a subsample with brain MRI data (*N* = 36,087), using multivariable linear regression.

**Results:**

Individuals with an inpatient diagnosis of peripheral vertigo had elevated risks of incident depression (hazard ratio (HR) 2.18; 95% confidence interval (CI) 1.79–2.67) and anxiety (HR 2.11; 95% CI 1.71–2.61), compared to others, particularly within 2 years after hospitalization (HR for depression 2.91; 95% CI 2.04–4.15; HR for anxiety 4.92; 95% CI 3.62–6.69). Depression was associated with lower FA in most studied white matter regions, whereas anxiety and peripheral vertigo did not show statistically significant associations with FA.

**Conclusions:**

Individuals with an inpatient diagnosis of peripheral vertigo have increased subsequent risks of depression and anxiety disorders, especially within 2 years after hospitalization. Our findings further indicate a link between depression and lower microstructural connectivity as well as integrity beyond the frontal-limbic network.

**Supplementary Information:**

The online version contains supplementary material available at 10.1186/s12916-023-03179-w.

## Background

Vertigo, defined as the illusion of movement, is a common symptom in the general population, especially the elderly [1]. Vertigo can be classified as peripheral or central vertigo, depending on dysfunctional locations in the vestibular pathway. Over 80% of vertigo is peripheral, with the most common causes including benign paroxysmal vertigo (BPV), Meniere’s disease (MD), vestibular neuritis (VN), and labyrinthitis. Vestibular information is transmitted to the cerebellum, vestibular nuclei, and cortex, where it is integrated with the visual and proprioceptive information to maintain the body balance. For instance, through nerve electrophysiological examination, it has been shown that the symptoms of vertigo can be directly caused by asymmetric activation of vestibular nuclei [2].

The comorbidity of peripheral vertigo and psychiatric disorders (e.g., depression and anxiety) is commonly reported in clinical practice. Peripheral vertigo usually presents with unpredictable, uncontrollable, and disabling attacks and is prone to relapse. People with peripheral vertigo can therefore be anxious and experience psychological distress. The reported prevalence of depression ranges from 6% to 50% whereas the prevalence of anxiety ranges from 3 to 50% in patients with vestibular dysfunction [3, 4]. Regardless, the existing evidence on the risk of clinically confirmed psychiatric disorders following peripheral vertigo is relatively weak due to methodological limitations, such as cross-sectional designs and small sample size, of the previous studies [5, 6].

A link between the vestibular and emotion processing systems is biologically plausible. One possible mechanism might be increased glucocorticoid levels, as consistent associations have been reported between vertigo, depression, and anxiety and increased cortisol levels [7, 8]. Another explanation might be altered connectivity in the frontal-limbic network. Neuroimaging studies have shown white matter abnormalities in the prefrontal cortex and limbic system among individuals with vestibular dysfunction, depression, or anxiety [9–11]. Electrophysiological studies have also suggested that the prefrontal cortex and hippocampus are involved in the processing of vestibular information [12]. However, no prior studies have investigated whether there is shared neural circuitry between peripheral vertigo and psychiatric disorders.

In the present study, we used the UK Biobank data to investigate whether peripheral vertigo is associated with increased subsequent risks of depression and anxiety disorders and to assess the involvement of altered neural circuitry in such association. We hypothesize that peripheral vertigo increases the risk of subsequent diagnosis of depression and anxiety, and that altered brain structural connectivity in the prefrontal–limbic network might underlie such association.

## Methods

### Study population

The UK Biobank is a prospective cohort study with over 500,000 participants aged 40–69 years when recruited between 2006 and 2010 throughout the UK. Details of the design of the UK Biobank are described elsewhere [13]. Briefly, all participants provided written informed consent; completed touchscreen questionnaire, verbal interview, physical examination, and biological sample collection at recruitment; and were continuously followed through repeated assessments and cross-linkages to national or regional health registers. Approximately 20,000 of them participated in the second assessment (during 2012–2013), 60,000 participated in the third assessment (during 2014–2019), and 5000 participated in the fourth assessment (since 2019). During the third assessment, participants underwent brain diffusion magnetic resonance imaging (MRI) examination.

**Fig. 1:**
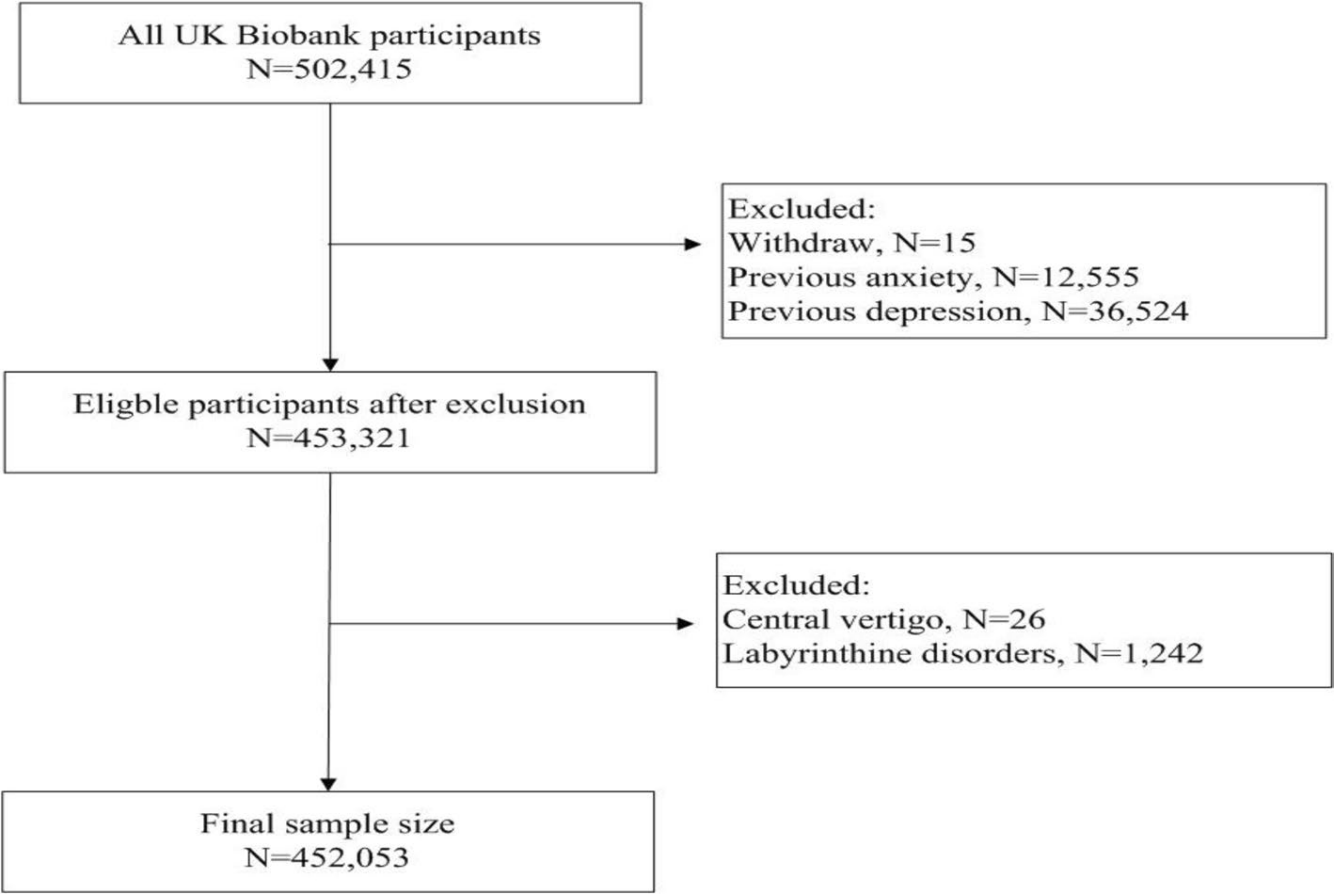
Flow chart of study population selection

Based on the UK Biobank data, we conducted a cohort study investigating the association between peripheral vertigo and subsequent risk of depression and anxiety disorders. We excluded 49,079 individuals with depression or anxiety before recruitment using combined first occurrence data from UKB (see below definitions for depression and anxiety) and 1268 individuals with central vertigo or labyrinthine disorders, leaving 452,053 individuals in the analysis (Fig. 1). We excluded participants with central vertigo to avoid the impact of brain tumor and stroke and excluded participants with labyrinthine disorders to avoid the impact of cochlear dysfunction. We followed the study participants from the date of recruitment until the first diagnosis of depression (or anxiety), loss to follow-up, death, or September 30, 2021, whichever came first.

An ethical approval of the UK Biobank was granted by the NHS National Research Ethics Service (reference number: 16/NW/0274), and all participants provided informed consent. This study was also approved by the Swedish Ethical Review Authority (DNR: 2022-01516-01).

### Peripheral vertigo

We identified hospitalization episodes for peripheral vertigo from inpatient medical records collected in the Hospital Episode Statistics in England, Patient Episode Database for Wales, and General Acute Inpatient and Day Case in Scotland, through the tenth version of the International Classification of Diseases (ICD) codes (Additional file 1: Table S1), using both the main diagnosis and secondary diagnoses of each hospitalization episode. We ascertained inpatient diagnosis of peripheral vertigo both before recruitment and during follow-up. Participants with peripheral vertigo before recruitment were defined as exposed from cohort entry, whereas participants with peripheral vertigo ascertained during follow-up were defined as unexposed until the diagnosis and exposed thereafter.

### Depression and anxiety

We identified the first occurrence of depression or anxiety using ICD-10 codes (Additional file 1: Table S1) from category 1712 in UK Biobank data. Details about the first occurrence data are described in “Resource 593.” Briefly, records from Primary Care (Category 3000, covering 45% of participants), hospital inpatient care (Category 2000, covering 100% of participants), Death Register (Fields 40001 and 40002, covering 100% of participants), and self-reported medical condition at the baseline or subsequent assessment center visits (Field 20002, covering 100% of participants) were all mapped to 3-character ICD-10 codes, and the earliest date of occurrence for each 3-character ICD-10 code was kept. During each assessment center visit, participants reported all health conditions diagnosed by a doctor and their answers were then verified with a nurse during the verbal interview (Field 20002). The diagnosis of depression has been validated and shown to have a positive predictive value of 73% for hospital inpatient care-based diagnosis [14], and 44% of the self-reported diagnosis were uniquely reported [15]. However, the diagnosis of anxiety has less concordance than depression between methods of ascertainment, indicating the need to incorporate data from multiple sources [16–18].

After excluding depression and anxiety before entering UKB, the incident depression and anxiety were defined from linkages with healthcare and register data (Category 3000, Category 2000, Fields 40001 and 40002) after recruitment to UKB and self-reported medical condition at subsequent assessment center visits (Field 20002).

### Brain MRI

Using the diffusion MRI data collected at the third assessment visit (during 2014–2019), we assessed the white matter fibers linking the medial frontal cortex, amygdala, hippocampus, and thalamus as well as the related vestibular pathways and main projection fibers [19] by fractional anisotropy (FA). FA is the measurement of water molecule diffusional directionality along fiber pathways and has been suggested as a biomarker in neuroimaging research of brain diseases. The post-processing details of these measurements have been described [20]. Based on standard procedure, FAs were extracted through tractography-based analysis in acoustic radiation, anterior/posterior/superior thalamic radiation, and the cingulate gyrus and parahippocampal part of the cingulum, as well as through tract-based spatial statistics in the corticospinal tract, inferior cerebellar peduncle, anterior/superior/posterior corona radiata, superior longitudinal fasciculus, uncinate fasciculus, cingulum cingulate gyrus, cingulum hippocampus, posterior thalamic radiata, and fornix cres+stria terminalis. We analyzed data in the left and right hemispheres separately.

### Covariates

As age, sex, and socioeconomic status might be associated with both peripheral vertigo and depression/anxiety [21, 22], we collected information on birth year, sex, educational attainment, and annual household income through touchscreen questionnaire at recruitment. Townsend deprivation index was calculated based on residential postcode recorded at recruitment and was used as a proxy of socioeconomic status.

### Statistical analysis

We compared characteristics of study participants according to the presence of hospitalization for peripheral vertigo before recruitment or during follow-up by ANOVA and chi-square tests.

We fitted multivariable Cox proportional hazards models to estimate hazard ratios (HRs) with 95% confidence intervals (CIs) of the associations between peripheral vertigo and subsequent diagnosis of depression and anxiety disorders. We performed two separate analyses, using the first diagnosis of depression and anxiety, respectively, as the outcome. The proportional hazard assumption was examined by plotting the Schoenfeld residuals over time and no major violation was found. We adjusted for age, sex, annual income, educational attainment, Townsend deprivation index, and assessment center. To investigate potential effect modification, we stratified the analyses by sex (male or female), age (≤ 50, 51–60, or > 60 years), educational attainment (college, tertiary, secondary, or below secondary), and annual income (≥ £31,000, £18,000~30,999, or < £18,000). We also calculated HRs and 95% CIs for different time periods (≤ 2, 2–5, or > 5 years) after an inpatient diagnosis of peripheral vertigo separately, by splitting the follow-up at 2 and 5 years since the start of follow-up to assess whether the studied associations would differ by time.

To assess the associations of depression, anxiety, and peripheral vertigo with the connectivity of specific brain regions, we fitted separate multivariable linear regression models for depression, anxiety, and peripheral vertigo, using participants with none of the three conditions as the reference group, with the adjustment for the same set of covariates as in Cox models.

Statistical analyses were conducted using the R software (version 4.0.2). We used the survival R-package version 3.4-0 function coxph to fit the Cox model, function cox.zph to test the proportional hazards assumption, and ggcoxzph to visualize the Schoenfeld residuals over time. We used the R-package survminer function ggcoxdiagnostics to test influential observations and ggcoxfunctional to test non-linearity. We found no major violations of the above assumptions. The statistical significance level was set at 0.05. All statistical tests were two-sided. In multivariable linear regression analyses, we used the Benjamini and Hochberg (BH) method to correct for multiple testing. We considered a false discovery rate (FDR) of < 0.05 as statistically significant.

## Results

Among the 452,053 participants, there were 53.2% female and the mean (SD) age at recruitment was 57.1 (8.1) years. A total of 2507 participants had an inpatient diagnosis of peripheral vertigo before recruitment (*N* = 387) or during follow-up (*N* = 2120), among whom 1048 had BPV, 1024 had MD, 204 had VN, and 230 had other peripheral vertigo (Table 1).

**Table 1:** Baseline characteristics of study participants according to status of peripheral vertigo

Characteristics	Total (*N* = 452,053)	No vertigo (*N* = 449,546)	Peripheral vertigo (*N* = 2507)				*p* value
			BPV (*N* = 1048)	MD (*N* = 1025)	Others (*N* = 230)	VN (*N* = 204)	
**Sex**							< 0.001
Female	240,550 (53.2)	239,038 (53.2)	672 (64.1)	612 (59.7)	124 (53.9)	104 (51.0)	
Male	211,503 (46.8)	210,508 (46.8)	376 (35.9)	413 (40.3)	106 (46.1)	100 (49.0)	
**Age at recruitment (years)**							< 0.001
Mean (SD)	57.1 (8.1)	57.1 (8.1)	61.1 (7.1)	60.3 (7.2)	59.9 (7.7)	59.0 (8.1)	
Range	37.4–73.7	37.4–73.7	40.3–70.2	40.4–70.1	41.0–70.0	40.5–70.1	
**Educational attainment**							< 0.001
College	146,495 (32.4)	145,901 (32.5)	239 (22.8)	254 (24.8)	57 (24.8)	44 (21.6)	
Tertiary	146,316 (32.4)	145,546 (32.4)	320 (30.5)	314 (30.6)	67 (29.1)	69 (33.8)	
Secondary	74,548 (16.5)	74,125 (16.5)	166 (15.8)	176 (17.2)	46 (20.0)	35 (17.2)	
Blow secondary	75,350 (16.7)	74,697 (16.6)	297 (28.3)	253 (24.7)	52 (22.6)	51 (25.0)	
Unknown	9344 (2.1)	9277 (2.1)	26 (2.5)	28 (2.7)	8 (3.5)	5 (2.5)	
**Annual income (pound)**							< 0.001
31,000 or more	202,912 (44.9)	202,151 (44.9)	303 (19.0)	334 (32.5)	57 (24.8)	67 (32.9)	
18,000 to 30,999	97,051 (21.5)	96,471 (21.5)	230 (21.9)	233 (22.7)	58 (25.2)	59 (28.9)	
Less than 18,000	82,593 (18.3)	81,921 (18.2)	292 (27.9)	264 (25.8)	72 (31.3)	44 (21.6)	
Unknown	69,497 (15.4)	69,003 (15.3)	223 (21.3)	194 (18.9)	43 (18.7)	34 (16.7)	
**Townsend deprivation index**							0.002
High	150,507 (33.3)	149,589 (33.3)	394 (37.6)	351 (34.2)	104 (45.2)	69 (33.8)	
Median	150,507 (33.3)	149,686 (33.3)	333 (31.8)	351 (34.2)	64 (27.8)	73 (35.8)	
Low	150,507 (33.3)	149,740 (33.3)	321 (30.6)	323 (31.5)	61 (26.5)	62 (30.4)	
Unknown	532 (0.1)	531 (0.1)	0 (0.0)	0 (0.0)	1 (0.4)	0 (0.0)	

*BPV* benign paroxysmal vertigo, *MD* Meniere’s disease, *VN* vestibular neuronitis, *Others*, other peripheral vertigo, other disorders of vestibular function, and disorders of vestibular function, unspecified

### Depression and anxiety

During a median follow-up of 7.8 years, a total of 17,064 and 15,912 participants were diagnosed with depression and anxiety disorders for the first time, respectively, among whom there were 98 cases (3.9%) of depression and 87 cases (3.5%) of anxiety in the group with peripheral vertigo (Table 2). Compared to other individuals, participants with an inpatient diagnosis of peripheral vertigo had higher risks of depression [HR 2.18; 95% CI 1.79–2.67; concordance = 0.616 (se = 0.002); Wald test = 2861 on 15 df, *p* = < 2e−16] and anxiety [HR 2.11; 95% CI 1.71–2.61; concordance = 0.611 (se = 0.002); Wald test = 2384 on 15 df, *p* = < 2e−16]. For depression, stronger associations were observed in males (HR 2.36; 95% CI 1.70–3.30), individuals over 60 years (HR 2.48; 95% CI 1.93–3.17), individuals with secondary (HR 2.45; 95% CI 1.56–3.85) or below secondary (HR 2.49; 95% CI 1.79–3.46) level of education, and individuals with medium income (£18,000~30,999; HR 2.38; 95% CI 1.58–3.58), compared to others. For anxiety, stronger associations were noted in females (HR 2.90; 95% CI 2.40–3.51), individuals over 60 years (HR 2.35; 95% CI 1.83–3.03), individuals with secondary (HR 2.86; 95% CI 1.86–4.40) or below secondary (HR 2.20; 95% CI 1.54–3.16) level of education, and individuals with an annual income below £18,000 (HR 2.39; 95% CI 1.69–3.36), compared to others. We found the highest risks of depression and anxiety within 2 years after an inpatient diagnosis of peripheral vertigo, with an HR of 2.91 (95% CI 2.04–4.15) for depression and an HR of 4.92 (95% CI 3.62–6.69) for anxiety (Table 3).

**Table 2:** Hazard ratio (HR) and 95 confidence interval (CI) for the association of peripheral vertigo with the risk of depression or anxiety

Groups	Depression		Anxiety	
	No. of cases among people with vertigo	HR (95% CI)	No. of cases among people with vertigo	HR (95% CI)
Overall				
	98	2.18 (1.79–2.67)	87	2.11 (1.71–2.61)
Sex				
Male	35	2.36 (1.70–3.30)	20	1.59 (1.03–2.47)
Female	63	2.06 (1.61–2.64)	67	2.90 (2.40–3.51)
Age at recruitment (years)				
~50	11	1.65 (0.91–2.97)	7	1.25 (0.60–2.63)
51~60	23	1.81 (1.20–2.73)	19	1.81 (1.16–2.85)
61~	64	2.48 (1.93–3.17)	61	2.35 (1.83–3.03)
Educational attainment				
College	13	1.56 (0.90–2.69)	16	2.09 (1.28–3.42)
Tertiary	28	2.15 (1.48–3.12)	19	1.53 (0.97–2.40)
Secondary	19	2.45 (1.56–3.85)	21	2.86 (1.86–4.40)
Below secondary	36	2.49 (1.79–3.46)	30	2.20 (1.54–3.16)
Annual income (pound)				
≥ 31,000	18	2.01 (1.26–3.19)	17	1.99 (1.23–3.20)
18,000~30,999	23	2.38 (1.58–3.58)	17	1.76 (1.09–2.83)
< 18,000	30	1.85 (1.29–2.65)	33	2.39 (1.69–3.36)
Unknown	27	2.68 (1.83–3.91)	20	1.98 (1.27–3.07)

Adjusted for age, sex, annual income, educational level, Townsend deprivation index, and assessment center. Hazard ratio for the “Unknown” group of educational attainment could not be calculated due to small sample size

**Table 3:** Hazard ratio (HR) and 95% confidence interval (CI) for the association of peripheral vertigo with depression or anxiety according to time since vertigo diagnosis

Time since vertigo diagnosis (years)	Depression		Anxiety	
	No. of cases among people with vertigo	HR (95% CI)	No. of cases among people with vertigo	HR (95% CI)
≤ 2	31	2.91 (2.04–4.15)	42	4.92 (3.62–6.69)
2 ~ 5	34	2.42 (1.72–3.39)	20	1.63 (1.05–2.53)
> 5	33	1.63 (1.16–2.30)	25	1.18 (0.80–1.75)

Adjusted for age, sex, annual income, educational level, Townsend deprivation index, and assessment center

### Brain white matter microstructure

**Fig. 2:**
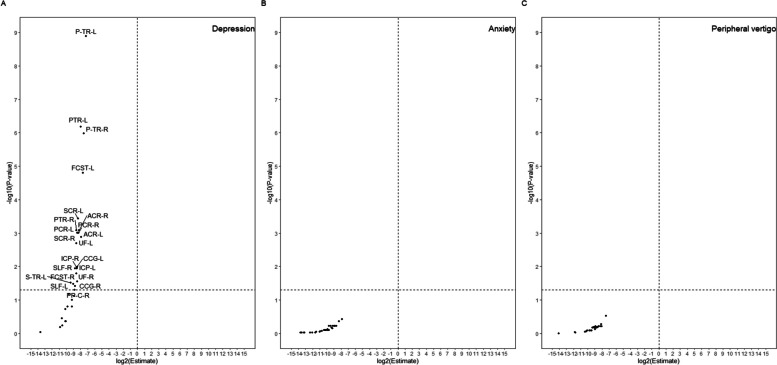
Connectivity of different brain regions in relation to depression, anxiety, and peripheral vertigo. Volcano plots show brain white matter connectivity in relation to depression, anxiety, and peripheral vertigo based on a linear regression after adjustment for age, sex, income, educational level, and Townsend index with no vertigo as reference. Horizontal dashed lines denote level of statistical significance with an FDR of 0.05. Vertical dashed lines denote no change of FA values (left: decrease; right: increase). ACR-L, anterior corona radiata left; ACR-R, anterior corona radiata right; SCR-L, superior corona radiata left; SCR-R, superior corona radiata right; PCR-L, posterior corona radiata left; PCR-R, posterior corona radiata right; PTR-L, posterior thalamic radiation on FA skeleton, left; PTR-R, posterior thalamic radiation on FA skeleton, right; UF_L, uncinate fasciculus left; FCST-L, fornix cres+stria terminalis left; CCG_L, cingulum cingulate gyrus left; P-TR-L, posterior thalamic radiation left; P-TR-R, posterior thalamic radiation right

We included 36,087 participants in this analysis, among whom 1139 were diagnosed with depression, 575 with anxiety, and 152 with peripheral vertigo. Compared to individuals with no vertigo, depression, or anxiety, individuals with depression had a lower FA in most studied white matter regions of interest (Fig. 2). In individuals with depression, lower FA was found in projection fibers, including the bilateral anterior/superior/posterior corona radiata, fornix cres+stria terminalis, the inferior cerebellar peduncle, and posterior thalamic radiata. Lower FA was also found in association fibers, namely the bilateral superior longitudinal fasciculus, which connects with the frontal cortex, uncinate fasciculus, and cingulum cingulate gyrus, as well as with different parts of the limbic system. Lower FA was also observed in the bilateral posterior thalamic radiation, left superior thalamic radiation, and right parahippocampal gyrus part of the cingulum.

Although individuals with anxiety or peripheral vertigo showed a tendency toward lower FA in the studied white matter regions, compared to individuals with no depression, anxiety, or peripheral vertigo, none of the differences was statistically significant (Fig. 2).

## Discussion

In this large community-based prospective cohort study, we found that peripheral vertigo, identified through an inpatient care episode, was associated with increased risks of subsequent diagnosis of depression and anxiety disorders, especially within 2 years after an inpatient diagnosis of peripheral vertigo. We further found that individuals with depression had lower FA in the fibers linking the frontal medial cortex, amygdala, hippocampus, and thalamus, compared to individuals without depression, anxiety, or peripheral vertigo.

### Peripheral vertigo and mental health

The present study showed that peripheral vertigo was associated with an increased risk of depression and anxiety disorders. Most previous studies show higher prevalence of depression and anxiety in patients with vestibular dysfunction, compared to healthy people, [3, 4] including one study suggesting that peripheral vertigo might be a risk factor for depression and anxiety [23]. The prevalence of depression and anxiety was lower in our study than reported in prior studies [3, 4]. This difference could be partially explained by the exclusion of individuals with depression or anxiety before recruitment as well as the different criteria used to ascertain depression and anxiety (i.e., we used ICD-10 codes and self-rating scales).

We quantified the risk of depression and anxiety over time since an inpatient diagnosis of peripheral vertigo and found that the risk for depression and anxiety was greatest during the first 2 years after such diagnosis and attenuated thereafter. This is in line with previous studies showing that the mental health of vertigo patients improved over time [5, 6, 24]. Specifically, two studies observed decreased symptoms of anxiety and depression during 3-year or 3-month follow-ups among patients with BPV and MD [5, 24] whereas one study reported that individuals diagnosed with MD long time ago had better mental health than those who were diagnosed recently [6]. Remission of acute symptoms after treatment [25] and improved understanding of vertigo might have reduced psychological distress, potentially explaining the reduced magnitude of the association with depression and anxiety later during follow-up. Regardless, the increased risk of depression and anxiety persisted 5 years after the start of follow-up, indicating a lasting susceptibility. Peripheral vertigo is usually self-limiting but may recur. Depression and anxiety disorders also fluctuate and, like vertigo, may respond to stress. Two studies have suggested that health anxiety induced by vestibular dysfunction, but not vestibular dysfunction itself, is associated with anxiety and depressive disorders, indicating that psychological factors may play an important role in the development of subsequent psychiatric disorders among patients with vertigo [26, 27]. Long-term surveillance on mood may therefore be considered in clinical practice.

### White matter microstructures

After the adjustment for potential confounders, including sex and age, we found that individuals with depression had a lower FA in a widespread brain network compared with the reference group. Our results extend previous knowledge by showing a more widely affected network including bilateral corona radiate and fibers involved in the vestibular pathway, as well as a more specific affected location in the cingulum.

The abnormalities in the frontal-limbic pathway and intra-limbic system among individuals with depression as noted in our study are in line with literature [28, 29]. Psychiatric neuroimaging studies have usually focused on prefrontal cortex and limbic areas, which are identified as key regions in the pathophysiology of mood disorders. FA alterations often occur in uncinate fasciculus and longitudinal fasciculus related to emotion and cognition. Harada et al. reported higher FA in the uncinate fasciculus in a study of 45 patients with depression, whereas Bhatia et al. reported significant reductions in FA in uncinate fasciculi of 103 patients with depression [28, 30]. The inconsistent results might be due to the use of different diagnostic criteria, scanners, and imaging sequences, as well as sample sizes between studies. The large-scale MRI data available in the UK Biobank allowed us to study a large population with the same diagnostic criteria and imaging sequences as well as to study more fibers such as corona radiata and vestibular pathway. The results showed lower FA in a more extensive network connecting prefrontal cortex, limbic system, thalamus, and vestibular nuclei. The entire corona radiata also demonstrated a lower FA. Such spreading white matter abnormalities might lead to dysfunction of vestibular cognition among individuals with depression. We also studied different parts of cingulum and found a lower FA in the anterior cingulum among patients with depression, which is responsible for processing emotion, not memory.

Neither anxiety nor peripheral vertigo was found to be significantly associated with declined structural connectivity or microstructural integrity in the studied white matter regions. Several explanations might underlie this result, taking into consideration the fact that we disregarded timing of the different events studied (i.e., peripheral vertigo/depression/anxiety vs. brain MRI measurement). First is *neuroplasticity in frontal-limbic pathways*. Although reduced structural connectivity of the frontal-limbic pathway has been suggested as a neuropathology basis for anxiety, [31, 32] anxiety treatment, either psychotherapy or pharmacotherapy, has been shown to reverse those alterations [11, 33]. Second is *limited understanding of vestibular pathways*. The vestibular system is a complex set of structures and neural pathways that serve various multisensory and sensorimotor functions. Recent studies have found several new integrative areas for vestibular signal processing, such as the thalamus and insular cortex, [34, 35] but the terminations of higher vestibular functions at the cortical level remain less known. Although we included the known fibers related to vestibular function in our study, there may be other relevant tracts. Third is *vestibular compensation*. Within the first few days after acute vestibular disturbance, vestibular compensation will begin to establish at multilevel in the vestibular pathway to reach rebalance. One of the key mechanisms is structural cortical plasticity in the multisensory vestibular-cortex, and this neuroplasticity would greatly reduce changes reflected in diffusion MRI data [36].

Although this finding could not support our hypothesis that the altered brain structural connectivity in the prefrontal–limbic network underlies the association between peripheral vertigo and depression/anxiety, it demonstrates neuroimaging characteristics of the white matter for these diseases that warrant future study. Studies on other mechanisms linking together peripheral vertigo and psychiatric disorders, such as cortisol regulation, are therefore still needed.

### Strengths and limitations

The strengths of the present study include the large-scale community-based study design, as well as the comprehensive data on exposure (peripheral vertigo) and outcomes (depression and anxiety). Another strength of the study is the performance of neuroimaging analyses in a large sample. All brain MRI data was assessed using standardized procedures and MRI protocols on dedicated scanners, and data cleaning was fully automated, largely limiting risks of subjective variations or errors. Our study has, however, some limitations. First, we identified only inpatient diagnosis of peripheral vertigo and therefore inevitably have misclassified participants who are healthier and have milder symptoms as unexposed which may result in underestimation of the studied associations. Related to this, the date of inpatient diagnosis of peripheral vertigo is believably later than the first clinical diagnosis. We therefore interpreted the temporal pattern of the studied association as by since first inpatient diagnosis of peripheral vertigo. Second, as we are primarily interested in newly onset depression and anxiety, we excluded participants with depression/anxiety before recruitment, focusing therefore on individuals that did not have depression/anxiety until a mean of 57.1 years of age. Therefore, the results are not directly generalizable to the general population with disparate susceptibility to depression/anxiety. Furthermore, although we examined depression and anxiety as two separate outcomes, around 30% of the individuals with depression/anxiety had indeed both diagnoses during follow-up, partly explaining perhaps the similar results observed for depression and anxiety. Third, we did not have primary-care diagnosis of depression and anxiety for all participants. Therefore, we supplemented the outcome definition with self-reported diagnosis in the attempt to capture as many cases with depression/anxiety as possible. Fourth, the timing of the brain imaging could be before or after the diagnosis of peripheral vertigo, depression, and anxiety. Therefore, a temporal order cannot be established among these events. However, the present study is aiming at investigating potential mechanisms explaining the link between peripheral vertigo and depression/anxiety rather than establishing causal events. Regardless, changes in the brain can happen already before the onset of symptoms for vertigo, depression, or anxiety, and persist after their diagnosis. Fifth, imaging data was not available for all UKB participants. Although participants with available brain imaging data are not a representative sample of the entire UK Biobank cohort, the participation in brain MRI exam was unlikely strongly related to vertigo or mental health [37, 38]. As a result, any selection bias related to this is unlikely to be substantial in our study. Lastly, although the UK Biobank has a large sample size, there were fewer than 100 participants with both peripheral vertigo and depression/anxiety in the present study. As a result, some of the analyses, especially the subgroup analyses, had insufficient power.

## Conclusions

In conclusion, we found that an inpatient diagnosis of peripheral vertigo was associated with an increased risk of depression and anxiety disorders, especially within 2 years after the inpatient diagnosis. Our findings also indicate that depression is associated with lower microstructural connectivity or integrity in the brain whereas neither anxiety nor peripheral vertigo showed such pattern.

### Supplementary Information

**Additional file 1:** **Table S1.** ICD-10 codes used to ascertain depression, anxiety, and peripheral vertigo.

## Data Availability

Data from the UK Biobank (https://www.ukbiobank.ac.uk/) are available to all researchers through application.

## Abbreviations

MRI: Magnetic resonance imaging
PTSD: Post-traumatic stress disorder
MD: Meniere disease
VN: Vestibular neuritis
BPV: Benign paroxysmal vertigo
FA: Fractional anisotropy
FMC: Frontal medial cortex
CG: Cingulate gyrus
AMY: Amygdala
HIP: Hippocampus
ICD: International Classification of Disease
