# Supplementary material for: Peripheral vertigo and subsequent risk of depression and anxiety disorders: a prospective cohort study using the UK Biobank
Source: BMC Med. 2024 Feb 9;22:63. doi: 10.1186/s12916-023-03179-w (PMC10858592; doi:10.1186/s12916-023-03179-w)
Supplement: Supplementary file 1 — Additional file 1: Table S1. ICD-10 codes used to ascertain depression, anxiety, and peripheral vertigo. [file 12916_2023_3179_MOESM1_ESM.docx]

**Additional File 1:**

**Table S1. ICD-10 codes used to ascertain depression, anxiety, and peripheral vertigo.**

| Diagnosis | ICD-10 codes |
| --- | --- |
| Depression | F32, F33 |
| Anxiety | F40, F41 |
| Peripheral vertigo  Central vertigo  labyrinthine disorders | H81.0, H81.1, H81.2, H81.3, H81.8, H81.9  H81.4  H83.0, H83.1, H83. |
